# Assessing the accuracy of computed tomography in detecting bony invasion and thickness of squamous cell carcinoma of the scalp

**DOI:** 10.1177/19714009211017777

**Published:** 2021-06-02

**Authors:** Conor T Boylan, Michaela S Gaston, Puja Merwaha, Kurdow Nader, Sukhbir Rayatt

**Affiliations:** 1 Medical School, University of Birmingham College of Medical and Dental Sciences, UK; 2 Queen Elizabeth Hospital, UK

**Keywords:** Computed tomography, squamous cell carcinoma, scalp tumours, depth assessment, bony invasion

## Abstract

**Objectives:**

The aim of this study was to ascertain the accuracy of computed tomography (CT) in assessing the presence of bony involvement and thickness of squamous cell carcinoma (SCC) of the scalp.

**Methods:**

A single-centre retrospective chart review was carried out. Inclusion criteria were scalp SCC, CT between January 2008 and 2018, and the availability of a reference test. Reference tests were either histology, surgical notes or clinical notes. Tabular assessment of accuracy was performed and Student’s *t*-test, Mann–Whitney U test and Fisher exact test were used in univariable analysis. Accuracy of thickness measurement was calculated using the limits of agreement method, and linear regression was used to examine trend.

**Results:**

Thirty-nine patients were included. Most patients were male (74.4%), white (97.4%), not immunosuppressed (66.6%) and had poorly differentiated tumours (33.3%). The most common tumour sites were the vertex (28.2%) and temporal region (23.1%). Sensitivity of CT in detecting presence or absence of bony invasion of scalp SCC was 76.9% (95% CI 46.2–94.9%) and specificity was 96.2% (95% CI 80.4-99.9%). Overall accuracy was 89.7% (95% CI 75.8–97.1%), positive predictive value was 90.1% (95% CI 58.7–99.8%) and negative predictive value was 89.3% (95% CI 71.8–97.7%). No significant differences were found comparing patients with an accurate or inaccurate CT scan. Thickness on CT was found to be consistent with histological thickness at the 95% confidence level.

**Conclusions:**

CT is accurate at assessing the presence of bony involvement and thickness of scalp SCC. This study was limited somewhat by small sample size.

## Background

The scalp is bounded anteriorly by the supraorbital margin, posteriorly by the superior nuchal line and laterally by the zygomatic arch.^1^ Deep to the scalp is the calvarium, which consists of a cortical outer table, a spongy marrow diploë and a second cortical inner table.^2^ To describe the zones of the scalp, this article will be using a previously defined nomenclature.^3^

Squamous cell carcinoma (SCC) is typically caused by ultraviolet sun damage and thus is a frequent occurrence on the oft-exposed face and scalp.^4,5^ It is the second most common cancer worldwide^6^ and is increasing in prevalence annually in the United Kingdom.^5^ SCC is locally invasive, has metastatic potential, and has a relatively high recurrence rate.^7,8^

Compared to skin SCC on other parts of the body, SCC occurring on the scalp appears to have a particularly poor prognosis.^9^ This is due to a number of synergistic factors including vertical growth limitation, dense vascularity, late discovery, ease of horizontal expansion, and close relation to the brain.^1,10–14^ The latter is particularly concerning, as extension of tumour beyond the inner table has a very poor prognosis; often being considered inoperable.^15,16^

Computed tomography (CT) scanning is the typical tool of choice to assess the thickness of scalp SCC and thus aid surgical planning and prognostication.^17^ There is, however, little data available to validate the use of CT scanning in this clinical setting. Numerous case reports describe CT being used to assess the depth of scalp SCC invasion,^12,13,16,18–23^ but only one cohort study exists that reports its accuracy, and this is limited by several important shortcomings.^24^ Validation of the use of CT for imaging bony invasion of scalp SCC would improve surgeon’s confidence when planning procedures and allow more accurate prognostication prior to and following surgery.

In this study we investigate the use of CT for assessing the presence of bony involvement and thickness of SCC of the scalp and postulate on its utility in this setting. We also examine the factors influencing the accuracy of CT in assessing the presence of bony involvement. We hypothesise that this study will find a high accuracy for CT scanning and support its use in clinical practice.

## Methods

This was a single-centre retrospective chart review study. Participants were extracted from a pre-formed database kept at a single tertiary NHS referral centre in the UK. All participants were included that had a diagnosis of primary SCC affecting the scalp, temple or forehead, were imaged with CT between January 2008 and 2018, and had a reference for confirmation of disease extent. Exclusion criteria were incomplete follow-up, confirmation of disease extent greater than 6 months from the time of the scan, incomplete medical records and previous cranioplasty at the site of the tumour. Owing to the rarity of this presentation, a formal power calculation was not performed; it was instead decided to simply include the maximum number of participants possible. References for disease extent were histological reports, explicit description of the presence or absence of bony invasion in the surgical notes or detection/exclusion of bony invasion in subsequent clinical notes (must have had at least 3 months recorded follow-up). These were chosen as they were easily accessible and were recorded as standard for most patients.

**Figure 1.**
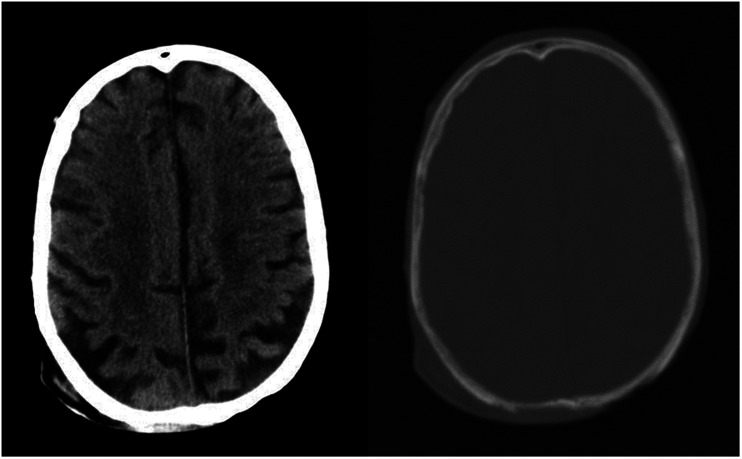
Contrast-enhanced CT head showing a large soft tissue defect over the right superior occipitoparietal area with marked thinning of the outer table of the underlying calvarium.

**Figure 2.**
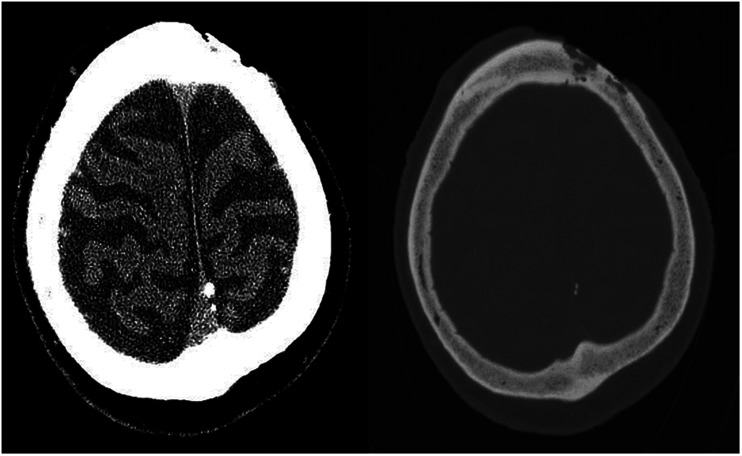
Non-enhanced CT head showing a localised skin defect in the left high frontal region with deep underlying bone erosion involving the outer and inner tables of the underlying calvarium. The permeative bone changes are multifocal and extend over approximately 3 cm diameter. No extension beyond the inner table is seen, with no evidence of involvement of the dura or brain parenchyma.

CT reports/images and medical notes were assessed for all participants. Baseline characteristics collected were date of birth, ethnicity, smoking status, significant comorbidities, immunocompromise and body mass index (BMI). Data collected from radiology reports were scan type, assessor, date of scan, use of contrast, slice thickness, presence of bony involvement and thickness of lesion. Images were additionally assessed for lesion thickness and presence of bony involvement by an independent reviewer. Bony invasion was defined as evidence of pathological thinning of the outer cortical table (Figure 1), through to full thickness erosive changes of both tables and diploë (Figure 2). Thickness was measured using the Fernandez–Flores method, following guidance from the Royal College of Pathologists.^25^

Details collected from histology reports were tumour type, date of assessment, presence of bony involvement, thickness, degree of differentiation and pathological stage. When the deep margin was involved, the thickness of the tumour was considered not to be assessable. A pathological stage of pT4 without the mention of perineural invasion of a named nerve was considered to indicate the presence of bony involvement.^25^ Where two grades of tumour differentiation were reported, the poorer grade was chosen for statistical analysis. Reviewers were not blinded to the results of the index or reference test.

Sensitivity, specificity, positive predictive values (PPVs), negative predictive values (NPVs), accuracy and positive and negative likelihood ratios (LR+ and LR− respectively) were calculated using Microsoft Excel for Office 365. 95% confidence intervals for sensitivity, specificity, PPV and NPV were calculated using the Clopper–Pearson exact method. All other statistical analysis was performed using the Statistical Package for Social Sciences 26 (SPSS26) software. Univariable analysis involved independent two-tailed Student’s *t*-tests to compare normally distributed continuous variables, Mann–Whitney U test for non-parametric continuous data and Fisher exact tests for nominal and ordinal data. Thickness as recorded on imaging was compared to thickness on histology using the limits of agreement method and displayed graphically with a Bland–Altman plot.^26^ Linear regression was used to assess for proportional bias and examine trend. *p*<0.05 was considered statistically significant throughout the study. An accurate scan was defined as one correctly identifying or correctly ruling out the presence of bony involvement.

Borders and zones of the scalp were defined using a previously described nomenclature dividing the scalp into seven distinct areas:^3^

- Forehead: the area between the anterior hairline superiorly, supraorbital ridge inferiorly, and temporal ridges laterally
- Frontal scalp: the area on the top of the head from roughly in line with the midpoint of the zygomatic arch to the anterior hairline
- Midscalp: the area between the frontal scalp and vertex, bordered laterally by the temporal/parietal hair fringes
- Vertex: a somewhat oval-shaped region in the posterior scalp overlying the lambda that is the typical site of the hair whorl in men
- Posterior scalp: the area overlying the occiput from vertex superiorly to superior nuchal line inferiorly and laterally to the mastoid portion of the temporal bone
- Temporal scalp: the lateral area of the head superior to the zygomatic arch and between the forehead and posterior scalp
- Posterior auricular area: from the posterior of the ears to the anterior margin of the posterior scalp.

This study was reviewed and approved by the local audit department. All patient data were collected as part of routine clinical practice, and patients had given informed consent for its use in medical research. Storage of data complied with the General Data Protection Regulation and the Data Protection act 2018.

Funding for this project came in the form of a £1100.00 research grant provided by the University of Birmingham as part of the Intercalated BSc Clinical Anatomy degree programme.

## Results

**Figure 3.**
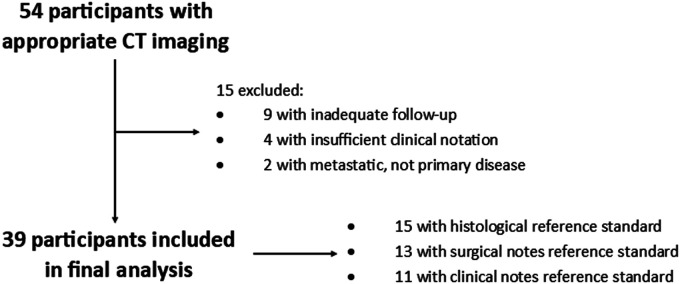
Diagram showing flow of participants through the study.

A total of 54 patients had appropriate CT imaging related to possible bony invasion of scalp SCC. Of these, 39 met the inclusion and exclusion criteria. Fifteen cases with appropriate imaging were excluded: nine were followed up for less than 3 months, four had insufficient clinical notation and two were metastatic SCC. Average time between scan and confirmation of disease extent was 0.8 months. Flow of participants is shown in Figure 3. No adverse events resulting from CT scan were recorded.

### Participant characteristics

Baseline characteristics for the whole cohort are outlined in Table 1. Fifteen (38.5%) patients had the extent of disease progression confirmed histologically, 13 (33.3%) were confirmed using the surgical notes and 11 (28.2%) were confirmed temporally using the clinical notes.

**Table 1.** Baseline characteristics for the whole cohort.

Characteristic	Whole cohort (*n*=39)
Age at imaging, years (SD)	74.9 (12.1)
Male (%)	29 (74.4)
Ethnicity (%)	
White	38 (97.4)
Asian	0
Black	0
Mixed ethnicity	0
Unknown	1 (2.6)
Smoking status (%)	
Never smoked	34 (87.2)
Current smoker	4 (10.3)
Ex-smoker	1 (2.6)
Immunosuppressed (%)	13 (33.3)
BMI, kg/m^2^ (SD)	26.7 (4.1)
Tumour location (%)	
Forehead	5 (12.8)
Frontal scalp	6 (15.4)
Midscalp	2 (5.1)
Vertex	11 (28.2)
Temporal region	9 (23.1)
Posterior auricular region	0
Posterior scalp	6 (15.4)
Tumour differentiation (%)	
Well	0
Moderate	11 (28.2)
Poor	13 (33.3)
Unknown	15 (38.5)
Previous scalp surgery (%)	19 (48.7)
Previous radiotherapy (%)	8 (20.5)

SD: standard deviation; BMI: body mass index.

### Assessment of bony invasion

CT results for presence of bony involvement are compared to the reference in Table 2. Eleven of the CT scans reported positive for bony invasion and 28 reported negative. Of those reporting a positive result, 10 were true positives. Of those reporting a negative result, 25 were true negatives. Using these values, sensitivity can be calculated as 76.9% (95% CI 46.2–94.9%), specificity as 96.2% (95% CI 80.4–99.9%) and overall accuracy as 89.7% (95% CI 75.8–97.1%). PPV can also be calculated as 90.1% (95% CI 58.7–99.8%) and NPV as 89.3% (95% CI 71.8–97.7%). LR+ is 20.0 and LR− is 0.2.

**Table 2.** Cross tabulation for accuracy of CT scan compared to the reference test (histology, surgical notes or temporal follow-up).

CT scan	Reference		
	Positive	Negative	Total
Positive	10	1	11
Negative	3	25	28
Total	13	26	39

Four patients had clear radiological evidence of intracranial involvement. There was complete agreement between radiology reports and assessment by the independent reviewer.

Baseline characteristics for patients in the *Accurate CT* cohort are compared to those for the *Inaccurate CT* cohort. Mean slice thickness for accurate CT scans was 1.2 mm (SD = 1.0 mm), compared to 1.6 mm (SD = 1.0 mm) for inaccurate scans; however, this did not reach statistical significance at the 95% confidence level (*p* = 0.064). No other statistically significant differences were found.

### Assessment of thickness

Mean lesion thickness across the whole CT cohort was 13.4mm (SD = 9.0 mm), but only 11 of these had histological confirmation of thickness, meaning the rest were excluded from statistical analysis.

**Figure 4.**
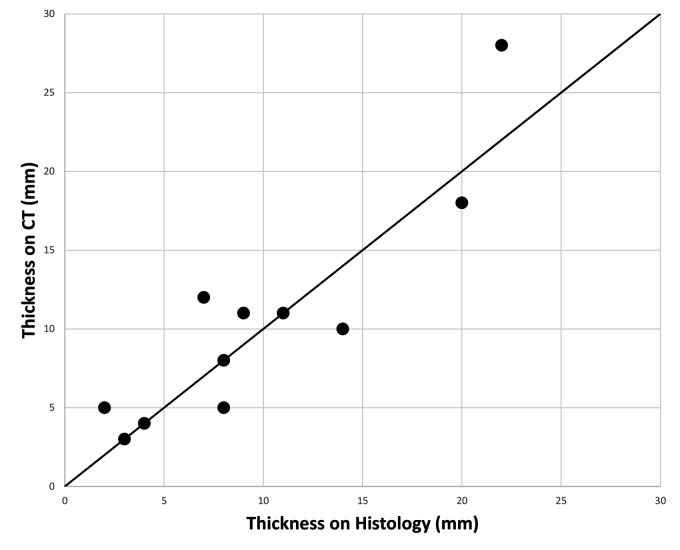
Scatter graph to show thickness on CT vs thickness on histology. Black line is line of no difference.

**Figure 5.**
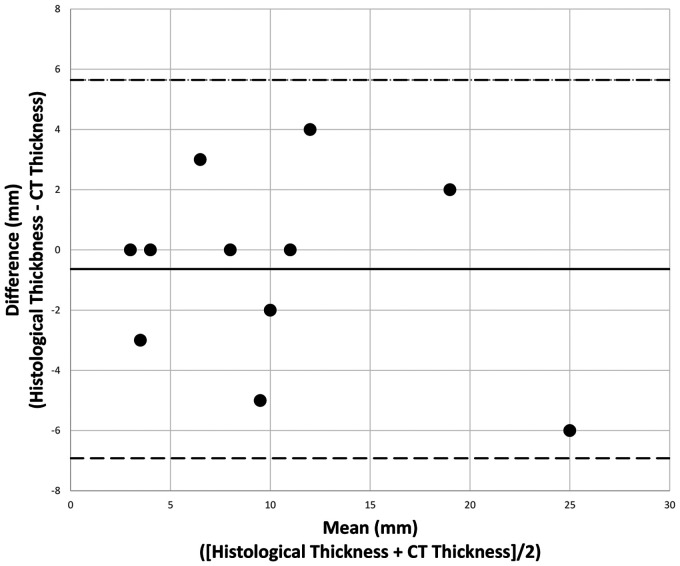
Bland–Altman plot showing difference between histological and CT thickness vs mean thickness across the two methods. Dashed horizontal lines are upper (upper line) and lower (lower line) limits of agreement at 95% confidence. Solid horizontal line is the mean difference.

For the 11 lesions with complete data, mean thickness on CT was 10.5 mm (± 7.3), compared to a mean thickness on histology of 9.9mm (± 6.5). This gives a mean difference of −0.6 m (± 3.1), with the limits of agreement being 6.2 mm. A scatter plot for thickness on histology vs thickness on CT is given in Figure 4. A Bland–Altman plot displaying the limits of agreement for the two methods is given in Figure 5. The Bland–Altman plot shows all values to fall within 1.96 standard deviations of the mean, indicating that the two methods correlate on their thickness measurements with a 95% confidence. Linear regression modelling echoed this, showing no proportional bias across the two tests (*B* = 0.113, *p* = 0.471).

## Discussion

In this retrospective chart review study of 39 patients, we found CT to have a high sensitivity (76.9%), specificity (96.2%) and overall accuracy (89.7%). Thickness as measured radiologically correlated with histological thickness at the 95% confidence level for the 11 lesions in which this data were available. The baseline characteristics for participants in this study were comparable to recent epidemiological data found in the literature.^5^

The sensitivity and specificity values of 76.9% and 96.2% respectively show CT to be a good imaging modality for both ruling in and ruling out the presence of bony invasion in SCC of the scalp. Positive and negative predictive values were also high (90.1% and 89.3% respectively), meaning conclusions drawn from a CT scan can be relied on most of the time. Overall accuracy was found to be 89.7% and LR+/LR− were found to be 20.0 and 0.2 respectively. This supports the use of CT in assessing bony invasion of SCC of the scalp.

This high accuracy is contrary to that reported by Kadakia et al.,^24^ who found sensitivity to be 30.0% and specificity to be 70.0% (LR+/LR− both 1.0). However, it is more consistent with robust systematic reviews assessing CT imaging of bony invasion in SCC of the oral cavity, for example in Bombeccari et al. where CT is reported to have a LR+ of 14.8 and LR− of 0.4.^27^ It is unclear why our findings differ so drastically from Kadakia et al.’s, but their study included only immunocompromised patients, with an average age 11.7 years younger than our cohort (63.2 years compared to our 74.9), and had a much more even male:female ratio (29:24, compared to 29:10 in our study). Kadakia et al. also did not investigate CT scan accuracy as a primary aim. The dearth of data makes it hard to interpret how these factors may have influenced findings, but it is clear that our studies have several key differences that make comparing results difficult.

One interesting point to note is the large number of patients who in fact did not have bony invasion present on the reference test. These patients made up 25/39 (64.1%) of cases. This implies that patients may be being over-referred for imaging. There is clearly benefit to ruling out the presence of bony invasion, but this needs to be contrasted with the risks posed by the radiation. Better-validated high-risk criteria may be useful in determining which patients truly need referral for imaging. This may also be an area that would benefit from more research in the future.

Univariable analysis was unable to identify any statistically significant impactors on the accuracy of CT scans, although a number of trends were noticed. The most significant finding here was that smaller slice thickness appeared to correlate at least somewhat with increased scan accuracy. It showed that the mean slice thickness in the *Accurate CT* cohort was 1.2mm compared to 1.6mm in the *Inaccurate CT* cohort (*p* = 0.064). While this association was not statistically significant at the 95% level, it fits very well with the published literature. Studies by Prionas et al. and Caivano et al. confirm that smaller slice thickness tends to correlate with greater scan accuracy, especially when tumours are relatively small, which is usually the case with SCC of the scalp.^28,29^

It was somewhat surprising that the use of contrast did not vary between groups, but uptake in the bony cortex is not typically very high, suggesting its effect on the ability to differentiate structures may be limited. The literature surrounding this is variable, with contrast being utilised frequently in bony invasion of oral SCC,^30,31^ but its usefulness being reported as variable in the calvarium.^32^

Mean CT lesion thickness for our study was 13.4mm (SD = 9.0 mm). This was in keeping with height measurements for scalp SCC as measured on MRI in a similar study.^33^ Using the limits of agreement method, it can be seen that the results of thickness assessment on CT are consistent with histological thickness at the 95% level. One possible explanation for the high correlation is the small cohort size and high variability in thickness measurements taken (SD = 7.3 mm on CT and 6.5 mm on histology). These factors widen the limits of agreement, resulting in a higher chance of concordance at the 95% level. This was accounted for somewhat using linear regression modelling, which demonstrated no significant trend in the results. Another explanation may be shrinkage of histological specimens during preparation as a result of intrinsic contraction.^34^ This could explain the slight overestimate (0.6 mm) that CT had on lesion size. However, given the available data, CT seems to be very good at assessing thickness of SCC of the scalp. Surgeons should be able to rely on it more confidently when planning procedures, as long as the small sample size (n = 11) is taken into account.

This study was limited somewhat by its small sample size, which came about due to the rarity of the presentation and the scarcity of adequate CT scans performed to assess it at our institution. This could be mitigated in future studies by pooling data from multiple establishments, subsequently increasing the number of participants. Another limitation was the imperfect reference standard for scan accuracy. Histology is the ideal comparator to measure accuracy against as it is carefully considered and gives accurate assessments of thickness, whereas surgical and clinical notes are subject to limitations including field of view, clinical experience and subjectivity. Again, this would have benefitted from a prospective study design or larger cohort size.

## Conclusions

This study demonstrated that CT was accurate at assessing the presence of bony involvement and thickness of SCC of the scalp. This accuracy may be improved by reducing slice thickness (*p* = 0.064) but does not appear to be affected by other parameters. In clinical practice the results of this study should be considered alongside the small study size and other limitations.
